# Identifying models of delivery, care domains and quality indicators relevant to palliative day services: a scoping review protocol

**DOI:** 10.1186/s13643-017-0489-4

**Published:** 2017-05-16

**Authors:** Seán R. O’Connor, Martin Dempster, Noleen K. McCorry

**Affiliations:** 1 Institute of Nursing and Health Research, Ulster University, Jordanstown, UK; 2 School of Psychology, Queen’s University Belfast, Malone Road, Belfast, UK; 3 Centre of Excellence for Public Health Northern Ireland, Centre for Public Health, School of Medicine, Dentistry and Biomedical Sciences, Queen’s University Belfast, Belfast, UK

**Keywords:** Palliative care, Quality indicators, Quality improvement, Systematic review

## Abstract

**Background:**

With an ageing population and increasing numbers of people with life-limiting illness, there is a growing demand for palliative day services. There is a need to measure and demonstrate the quality of these services, but there is currently little agreement on which aspects of care should be used to do this. The aim of the scoping review will be to map the extent, range and nature of the evidence around models of delivery, care domains and existing quality indicators used to evaluate palliative day services.

**Methods:**

Electronic databases (MEDLINE, EMBASE, CINAHL, PsycINFO, Cochrane Central Register of Controlled Trials) will be searched for evidence using consensus development methods; randomised or quasi-randomised controlled trials; mixed methods; and prospective, longitudinal or retrospective case-control studies to develop or test quality indicators for evaluating palliative care within non-residential settings, including day hospices and community or primary care settings. At least two researchers will independently conduct all searches, study selection and data abstraction procedures. Meta-analyses and statistical methods of synthesis are not planned as part of the review. Results will be reported using numerical counts, including number of indicators in each care domain and by using qualitative approach to describe important indicator characteristics. A conceptual model will also be developed to summarise the impact of different aspects of quality in a palliative day service context. Methodological quality relating to indicator development will be assessed using the Appraisal of Indicators through Research and Evaluation (AIRE) tool. Overall strength of evidence will be assessed using the Grading of Recommendations, Assessment, Development, and Evaluation (GRADE) system. Final decisions on quality assessment will be made via consensus between review authors.

**Discussion:**

Identifying, developing and implementing evidence-based quality indicators is critical to the evaluation and continued improvement of palliative care. Review findings will be used to support clinicians and policymakers make decisions on which quality indicators are most appropriate for evaluating day services at the patient and service level, and to identify areas for further research.

**Electronic supplementary material:**

The online version of this article (doi:10.1186/s13643-017-0489-4) contains supplementary material, which is available to authorized users.

## Background

With an ageing population and increasing numbers of people with life-limiting illness, there is a growing demand for palliative care day services. Consequently, there is a need to measure the quality and value of these services, but there is little agreement on which aspects of care should be used to do this. Previous studies have indicated that day services may be associated with high patient satisfaction, but there is currently limited evidence demonstrating that services lead to significant improvements in symptom control or quality of life [1, 2]. Palliative day services have also been shown to differ substantially across settings, with wide variations in staffing structures, core components of care and models of delivery [3, 4]. Evaluating quality of palliative day services is essential for assessing care across diverse settings and for monitoring quality improvement approaches. Development and implementation of quality indicators for palliative day services is therefore a key priority.

Recent studies have used established methods to develop quality indicators in general palliative care [5, 6], but there is evidence to suggest that separate indicators may be required in different contexts, such as non-residential settings including day hospice, community or primary care settings [7]. Since quality of care is a multidimensional concept, its assessment can require different measures. Indicators are not a direct measure of quality but are centred on evidence-based standards of care. In this manner, indicators can be used as quantitative measures to assess care processes, structures or outcomes and to monitor, evaluate and improve the quality of important clinical and support functions, as well as governance and management factors that can influence patient outcomes [8]. Consequently, they allow for comparisons to be made over time and across settings and to set priorities and support patient choice. It is important to recognise that process and structural indicators can only be considered as valid when it can be demonstrated through high-quality evidence that they result in improved outcomes. These associations can derive from primary research, as well as reviews of existing evidence and expert consensus methods.

This planned review will inform the initial phases of the UK Consensus Project on Quality in Palliative Care Day Services, a project which aims to develop an evidence-based quality indicator set for day services using the multiphase RAND/UCLA appropriateness method [9].

### Objectives

The aim of this scoping review will be to map the extent, range and nature of the existing evidence around models of delivery, care domains and quality indicators used to evaluate palliative day services. In doing so, the review will also seek to detect gaps in this evidence and identify future directions for research in the area. Specific objectives will be (1) to identify existing quality indicators relevant to palliative day services and to evaluate the quality of their development and (2) to categorise existing quality indicators by care domain and model of delivery.

## Methods

A scoping review methodology will be employed as these approaches take account of a broader range of study designs and methods than systematic reviews, which typically address research questions around the effectiveness of a specific intervention. Established methodological frameworks and recommendations will be used to guide the review process [10–12]. The review will include the following components or phases: (1) formulation of research questions, (2) identification and selection of relevant evidence, (3) extraction of potential quality indicators from existing evidence, (4) charting and synthesis of results including development of a conceptual model and (5) engagement and consultation with key stakeholders to ensure relevance of the conceptual model to current practice and to facilitate knowledge exchange processes. In addition to the use of this methodological framework, the Preferred Reporting Items for Systematic reviews and Meta-Analyses for Protocols (PRISMA-P) checklist [13] will be followed to ensure preparation of a robust protocol (see Additional file 1).

### Search strategy

A systematic search strategy will be carried out by combining key Medical Subject Heading (MeSH) terms including “Quality indicator”, “Patient-centered care”, “Quality criteria” and “Quality improvement”. The proposed MEDLINE search strategy is shown in Table 1. Key search terms will be identified though discussion between project team members and with other experts and stakeholders in the area. The fully developed systematic search strategy will be peer reviewed using the Peer Review of Electronic Search Strategies (PRESS) tool [14]. Five electronic databases (Ovid MEDLINE via Ovid, EMBASE via Ovid, CINAHL via EBSCO, PsycINFO and the Cochrane Central Register of Controlled Trials) will be searched from January 2000 to the present day. Only evidence published since 2000 will be included to ensure relevance to current palliative care settings and day service delivery models. No language restrictions will be applied during searches. Hand searches of the reference lists from included articles and relevant systematic reviews will also be carried out. The search strategy will also use previous recommendations, which are based on a feasible and robust method of applying a systematic search to identify grey literature [15]. Grey literature searches will be conducted using http://www.opengrey.eu/ and Google Scholar to identify projects in progress, practice guidelines and policy documents or reports not indexed in the electronic databases. Google Trends (https://www.google.com/trends/), a publically accessible resource providing data on geospatial and temporal patterns for user-specified terms, will be used to identify closely matching terms commonly used to locate information around quality of care. Given the broad scope of the review, only the first 50 returns from the website searches will be extracted. The websites of ten key palliative care and general healthcare organisations will also be searched.

**Table 1:** Proposed MEDLINE search strategy developed using Medical Subject Headings (MeSH)

Search strategy
1 QUALITY INDICATOR$.mp.
2 exp Quality Indicators, Health Care/og, st, ut [Organization & Administration, Standards, Utilization]
3 exp QUALITY INDICATOR$, HEALTHCARE/
4 PATIENT-CENTERED CARE/
5 exp “Standard of Care”/og, st [Organization & Administration, Standards]
6 Quality Assurance, Health Care/mt, og, st [Methods, Organization & Administration, Standards]
7 QUALITY CRITERIA.mp.
8 QUALITY IMPROVEMENT.mp. or Quality Improvement/
9 QUALITY OF HEALTH CARE/
10 PROCESS ASSESSMENT HEALTH CARE.mp. or “Process Assessment(Health Care)”/
11 GUIDELINES AS TOPIC/
12 PRACTICE GUIDELINES AS TOPIC/
13 exp Practice Guideline/
14 BEST PRACTICE/
15 PALLIATIVE CARE/
16 HOSPICE PROGRAMS.mp. or exp Hospice Care/
17 HOSPICE.mp. and PALLIATIVE CARE NURSING/[mp=ti, ab, hw, tn, ot, dm, mf, dv, kw, nm, kf, px, rx, ui, tc, id, tm]
18 "Quality of Life"/or PALLIATIVE MEDICINE.mp. or exp Palliative Medicine/
19 DAY CARE/
20 REHABILITATION/
21 Complementary Therapies/or THERAP$, COMPLEMENTARY.mp.
22 THERAPY, RELAXATION/
23 exp Relaxation/px, tu [Psychology, Therapeutic Use]
24 INTRAVENOUS INFUSIONS/
25 INTRAVENOUS INFUSION$.mp. or exp Infusions, Intravenous/
26 MANAGEMENT, PAIN/
27 exp Pain Management/mt, nu, px, st [Methods, Nursing, Psychology, Standards]
28 SUPPORT SYSTEM, PSYCHOSOCIAL/
29 DELPHI TECHNIQUE$.mp.
30 CONSENSUS DEVELOPMENT.mp.
31 Q SORT.mp.
32 RAND APPROPRIATENESS METHOD.tw.
33 RAND APPROPRIATENESS.mp.
34 1 or 2 or 3 or 4 or 5 or 6 or 7 or 8 or 9 or 10
35 11 or 12 or 13 or 14
36 15 or 16 or 17 or 18
37 20 or 21 or 22 or 23 or 24 or 25 or 26 or 27 or 28
38 29 or 30 or 31 or 32 or 33
39 34 and 36
40 19 and 34
41 34 and 37
42 34 and 35
43 34 and 38
44 limit 39 to human
45 limit 44 to yr=“1990 -Current”
46 limit 40 to human
47 limit 46 to yr=“1990 -Current”
48 limit 41 to human
49 limit 48 to yr=“1990 -Current”
50 limit 42 to human
51 limit 50 to yr=“1990 -Current”
52 limit 43 to human
53 limit 52 to yr=“1990 -Current”

### Study screening and inclusion

Two researchers (SOC, MD) will independently conduct the database searches and import all potential evidence sources into an EndNote referencing software database (EndNote X7.3, Thomson Reuters, Ontario, Canada). The same researchers will then screen the title and abstract of records identified during the searches to assess relevance of the content in comparison with the above eligibility criteria. Non-relevant titles will be removed at this stage. Full-text copies of potentially relevant articles will be obtained and reviewed to determine inclusion using the same criteria. Agreement between reviewers at this stage will be determined using the κ statistic with an accepted cut-off point of above 0.60 used to indicate adequate agreement [16]. A third reviewer will be used to resolve any disagreement or uncertainty around final inclusion (NMcC). All evidence excluded during screening will be recorded, along with the reason for exclusion. Reviewers will not be masked to author name or publication type during the screening process.

### Study eligibility criteria

To broaden the scope of the review and reflect the diverse nature of day services, the review will include all evidence identified which involved the development or evaluation of quality indicators to assess palliative care within any non-residential setting, including day hospice and community or primary care settings. The review will focus on quality indicators for adults receiving care for any life-limiting conditions including cancer or uncontrolled symptoms due to cancer treatment, heart failure, pulmonary disease, dementia, end stage liver or renal disease and neurologic conditions including multiple sclerosis, motor neurone disease or Parkinson’s disease. We will not include indicators developed to assess care in patients undergoing surgical procedures, as measures of quality and care pathways are distinct in this group of patients. The following types of evidence will be considered: consensus development methods; randomised or quasi-randomised controlled trials; mixed methods; prospective, longitudinal or retrospective case-control studies. Clinical practice guidelines, consensus statements, systematic reviews and relevant reports or policy documents will also be included. Where indicators are identified from these latter sources, the review will attempt to ascertain the original development research. All included evidence will be required to identify or propose at least one potential quality indicator related to structures, processes or outcomes at the individual patient or service level of care. Quality indicators will be defined as any measure, able to be expressed as a fraction, which compares actual care against an ideal criterion [17, 18]. The review will therefore include not only evidence directly identifying candidate indicators but also evidence that discusses the outcome or event of interest without proposing the event as a quality indicator.

### Data extraction

Two researchers (SOC, MD) will independently use a standard data abstraction form to chart the following information from the included evidence: author and date, country, document type (research paper, guideline, policy document etc.), target population/setting, indicator sets (domains), methods used in development, proposed methods of measurement and evidence of testing or implementation. Specific information on numerator and denominator descriptions, definitions and type of quality indicators will also be extracted and classified as structural, process or outcome indicators using the Donabedian Framework [19]. According to this model, structural indicators include factors that affect the context in which care is delivered (i.e. organisational characteristics such as availability of equipment and services or staffing). Process indicators relate to the delivery of care (i.e. assessment and interventions), while outcome indicators are concerned with the effectiveness of care in terms of change in patient important outcomes (i.e. health status, satisfaction with care and overall quality of life). Indicators removed at each phase will also be verified by all authors.

The data abstraction form will be piloted using a sample of ten non-relevant quality indicator articles in order to standardise the abstraction process and make any required modifications. The quality of each indicator will be assessed by all reviewers using the Appraisal of Indicators through Research and Evaluation (AIRE) tool, which uses a combined score based on 20 items across four domains: purpose, relevance and organisational context; stakeholder involvement; scientific evidence, and additional evidence, formulation and usage [20]. Each item will be rated on a 4-point Likert scale ranging from “1” (strongly disagree: confident that the criterion has not been fulfilled or no information was available) to “4” (strongly agree: confident that the criterion has been fulfilled). The mean score, reported as a percentage, will be determined for each domain, as well as for the overall score assigned. Indicators will be considered to be of good quality if they score 50% or more in all four domains. The quality of overall evidence will be assessed initially by two reviewers (SOC, MD) using the Grading of Recommendations, Assessment, Development, and Evaluation (GRADE) system [21]. This system appraises and summarises the quality and strength of recommendations across important outcomes. Overall evidence for each outcome will be rated as very low, low, moderate or high quality, based on study design and limitations, consistency of effect and the directness or generalisability of evidence [22]. Final decisions on methodological quality will be reached through consensus between review authors. While appraisal of methodological quality is not considered as essential during conduct of a scoping review [23], the assessments planned as part of this review will provide valuable information for the evidence summary tables that will be produced for the initial phases of the RAND/UCLA appropriateness method [9].

All quality indicators extracted from the evidence sources, including their original descriptions, will be combined in a single excel file (Microsoft, Washington, USA). This list will then be scrutinised and non-relevant or duplicate indicators will be removed. The original and revised file will be reviewed and cross checked by a second reviewer, with any disagreement or uncertainty resolved via discussion with a third reviewer. The final list of quality indicators will be agreed by consensus among the reviewers. The indicators will then be grouped according to their proposed quality domain. These domains will be determined based on those used in previous quality indicator development projects identified during the review and following discussion with the review team (see Tables 2 and 3).

**Table 2:** Proposed quality domains for the project

QD#01: Physical care and support, assessment and treatment
QD#02: Psychological care and support, assessment and treatment
QD#03: Social care and support, assessment and treatment
QD#04: Spiritual and emotional care and support
QD#05: Cultural aspects of care
QD#06: Generic aspects of care and health promotion
QD#07: Information and communication with patients, carers and family
QD#08: Care planning, goal setting and shared decision making with patients, carers and family
QD#09: End of life care and decisions
QD#10: Pre and post-bereavement support
QD#11: Co-ordination and continuity of care
QD#12: Structure and process of care
QD#13: Evidence of effectiveness, outcome assessment and measurement
QD#14: Staff training and education, service and professional development
QD#15: Access to services and service environment
QD#16: Promotion of effective external engagement
QD#17: Societal, ethical and legal aspects of care

*QD* quality domain

**Table 3:** Quality domains used in key clinical guidelines, quality indicator development projects and systematic reviews

Author and year	Country/area^a^	Population	Total number of quality domains (indicators or recommendations)	Quality domains
van der Steen JT et al. European Association for Palliative Care (EAPC). White paper defining optimal palliative care in older people with dementia: a Delphi study and recommendations from the European Association for Palliative Care. Palliat Med. 2014 Mar;28(3):197–209.	Europe	Older adults with dementia	11 [57]	Applicability of palliative care Person-centred care, communication and shared decision making Setting care goals and advance planning Continuity of care Prognostication and timely recognition of dying Avoiding overly aggressive, burdensome or futile treatment Optimal treatment of symptoms and providing comfort Psychosocial and spiritual support Family care and involvement Education of the health care team Societal and ethical issues
Uphoff EP et al. Development of generic quality indicators for patient-centred cancer care by using a RAND modified Delphi method. Cancer Nurs. 2012 Jan-Feb;35(1):29-37.	Holland	Adults with cancer	5 [17] QIs developed from 92 initial recommendations	Communication Physical support Psychosocial care After-care Organisation of care
Leemans K et al. Towards a standardised method of developing quality indicators for palliative care: protocol of the Quality indicators for Palliative Care (Q-PAC) study. BMC Palliat Care. 2013 Feb 8;12:6.	Belgium/Holland	Palliative care	9 [53]	Assessment and treatment of symptoms Assessment and treatment of psychological problems Assessment and treatment of spiritual and religious problems Informing patients and their family members Care planning with patients and family members Treatment and end of life decisions Type of care and circumstances of dying Family support Continuity of care
National Consensus Project for Quality Palliative Care, Clinical Practice Guidelines for Quality Palliative Care, 2013	USA	Palliative care	8 [27]	Structure and processes of care Physical aspects of care Psychological and psychiatric aspects of care Social aspects of care Spiritual, religious and existential aspects of care Cultural aspects of care Care of the imminently dying patient Ethical and legal aspects of care
National Quality Forum: A National Framework and Preferred Practices for Palliative and Hospice Care Quality. Washington, DC, National Quality Forum, 2006	USA	Palliative care	8 [33]	Structures and processes of care Physical aspects of care Psychological and psychiatric aspects of care Social aspects of care Spiritual, religious and existential aspects of care Cultural aspects of care Care of the imminently dying patient Ethical and legal aspects of care
McCusker M et al. Institute for Clinical Systems Improvement. Palliative Care for Adults. Updated November 2013.	USA	Palliative care	5 [13]	Patient presents with new or established diagnosis of a serious illness^b^ Initiate palliative care discussion^b^ Assess patient’s palliative care needs Physical aspects of care Cultural aspects of care Psychological and psychiatric aspects of care Social aspects of care
De Roo ML et al. Quality indicators for palliative care: update of a systematic review. J Pain Symptom Manage. 2013 Oct;46(4):556-72.	EURO IMPACT Belgium/Holland	Palliative care	8 [33]	Structure and process of care Physical aspects of care Psychological and psychiatric aspects of care Social aspects of care Spiritual, religious and existential aspects of care Cultural aspects of care Care of the imminently dying patient Ethical and legal aspects of care
Mularski RA et al. Proposed quality measures for palliative care in the critically ill: a consensus from the Robert Wood Johnson Foundation Critical Care Workgroup. Crit Care Med 2006;34(11 Suppl):S404eS411.	USA	Palliative care	7 [18]	Patient and family-centred decision making Communication within the team and with patients and family Continuity of care Emotional and practical support for patients and family Symptom management and comfort care Spiritual support for patients and family Emotional and organisational support for clinicians
Dy SM et al. Measuring what matters: top-ranked quality indicators for hospice and palliative care from the American academy of hospice and palliative medicine and hospice and palliative nurses association. J Pain Symptom Manage. 2015 Apr;49(4):773-81	USA	Palliative care	9 [10]	Structure and processes of care Physical aspects of care Psychological and psychiatric aspects of care Social aspects of care Spiritual, religious and existential aspects of care Cultural aspects of care Care of the patient at the end of life Ethical and Legal Aspects of care Global measures
National Institute of Clinical Excellence (NICE). 2004. Guidance on Cancer Services Improving Supportive and Palliative Care for Adults with Cancer. The Manual.	UK	Palliative care	12 [20]	Co-ordination of care User involvement Face-to-face communication Information Psychological support services Spiritual support services General palliative care services, including care of dying patients Specialist palliative care services Rehabilitation services Complementary therapy services Services for families and carers, including bereavement care Workforce development
van Riet Paap J et al. IMPACT research team. Consensus on quality indicators to assess the organisation of palliative cancer and dementia care applicable across national healthcare systems and selected by international experts. BMC Health Serv Res. 2014 Sep 17;14:396.	Holland	Palliative cancer and dementia care	7 [23]	Access to palliative care Infrastructure Assessment tools Personnel Documentation of clinical data Quality Education
Vasse E et al. The development of quality indicators to improve psychosocial care in dementia. Int Psychogeriatr. 2012 Jun;24(6):921-30.	Holland	Dementia	4 [12]	Diagnosis and assessment Care plan and treatment Behavioural problems Caregivers
Woitha K et al. Validation of quality indicators for the organization of palliative care: a modified RAND Delphi study in seven European countries (the Europall project). Palliat Med. 2014 Feb;28(2):121-9.	Holland (Europal)	Palliative care	6 [56]	Definition of a palliative care service Access to palliative care Assessment tools Personnel in palliative care services Quality and safety Reporting clinical activity of palliative care services

*NICE* National Institute for Clinical Excellence

^a^Based on first author location

^b^Not included as domains

### Data synthesis

Due to the predicted breadth and heterogeneity of the identified evidence, meta-analyses and statistical methods of synthesis are not planned as part of the review. Quantitative results will be reported using numerical counts, including year of publication, country, evidence type and the number of indicators in each care domain. A qualitative approach will be used to describe indicator characteristics in terms of their method of reporting, evidence of reliability testing and the methodological quality of their development. All data processing, including calculation of the κ statistic to determine reliability of the search strategy, will be handled using SPSS (Version 21.0). Extracted information will be used to develop plain language evidence summary tables, with quantitative data displayed in graphical form. These will be used to guide the expert rating phase using the RAND/UCLA appropriateness method [9]. To further aid the expert rating process, a concise and standardised list of indicator definitions will be developed by using a priori criteria to standardise the wording of the indicators in the evidence summary tables. For example, where indicator definitions include any IF and THEN statements, or reference to a specified condition, these will be removed. Any mention of exact timing (e.g. how many days before an intervention is started after referral) will also be removed as it is expected that this will differ for day services in comparison to other care settings. Since it is anticipated that some indicators will be generally comparable but may be measured in a number of ways or used in different care settings, the extracted information will be also used to develop a conceptual model, based on the Donabedian framework [18], and a modified version of the framework used in the OECD Health Care Quality Indicators Project [24]. The model will summarise the impact of different aspects of quality in a palliative day care context and will help to identify areas of discussion among the expert panel during the consensus meeting phase of the RAND/UCLA appropriateness process [9]. This model will also be used to identify gaps in the evidence or areas where indicators are not currently available to evaluate existing services. Particular focus will be placed on the context in which certain indicators have been developed or implemented to ensure relevance to current practice (see Additional file 2).

## Discussion

Identifying, developing and implementing evidence-based quality indicators is critical to the evaluation and continued improvement of palliative care. The planned scoping review will form part of a larger research process aimed at developing capacity in the evaluation and implementation of quality indicators in palliative day services. The review is intended to reflect and effectively summarise the diverse nature of palliative care delivered in day hospice, community or primary care settings. It is anticipated that a substantial number of indicators, distributed across different areas and models of care will be identified. Key stakeholders involved in the overall project (including hospice and day service leads, medical and nursing staff, healthcare professionals (physiotherapists, occupational therapists, complementary therapists, psychologists), volunteer staff and service users) will also be included in the summarising and reporting phase of the research process. Feedback provided by relevant stakeholders at this stage will be used to ensure relevance to current practice and to promote knowledge exchange and translation processes. The review findings will be used to identify gaps in the current evidence and to inform a planned systematic review. Findings will also be used to assist clinicians and policymakers ascertain whether existing indicators are appropriate for evaluating palliative day services at the patient and service level and to categorise how indicator measurement and quality improvement approaches fit within existing models of care.

## Additional files

Additional file 1: PRISMA-P checklist. Checklist including list of recommended items to include in a systematic review protocol. (29.7 Kb)

Additional file 2: Proposed conceptual model mapping quality indicators by stage of care. A proposed conceptual model based on the Donabedian framework and a modified version of the framework used in the OECD Health Care Quality Indicators Project. (21.7 Kb)

## Abbreviations

AIRE: Appraisal of Indicators through Research and Evaluation tool
GRADE: Grading of Recommendations, Assessment, Development, and Evaluation
MeSH: Medical Subject Heading
PRESS: Peer Review of Electronic Search Strategies
PRISMA-P: Preferred Reporting Items for Systematic reviews and Meta-Analyses for Protocols
