# Supplementary material for: Identifying models of delivery, care domains and quality indicators relevant to palliative day services: a scoping review protocol
Source: Syst Rev. 2017 May 16;6:100. doi: 10.1186/s13643-017-0489-4 (PMC5434637; doi:10.1186/s13643-017-0489-4)
Supplement: Supplementary file 2 — Proposed conceptual model mapping quality indicators by stage of care. A proposed conceptual model based on the Donabedian framework and a modified version of the framework used in the OECD Health Care Quality Indicators Project. (21.7 Kb) [file 13643_2017_489_MOESM2_ESM.docx]

**Additional Figure 1. Proposed conceptual model mapping quality indicators by stage of care***

| **Population** | Adults (and their carers / family) attending day services with any life limiting condition including: Cancer; Heart failure; Pulmonary disease; Dementia; End stage liver or renal disease; Multiple sclerosis; Motor neurone disease; Parkinson’s disease |
| --- | --- |

| **Stage of care** | **Number of indicators** | **Main focus of care at each stage** | **Indicator type[s]**  **Structure , Process, Outcome** |
| --- | --- | --- | --- |
| Access and referral, resources and staffing |  | ● Equity of access and referral, availability of comprehensive services and suitable equipment  ● Continuing staff education | ● |
| Triage, screening and assessment |  | ● Screening for main physical and psychosocial symptoms and complaints  ● Assessment of important spiritual or emotional aspects of care | ● |
| Care planning and shared decision making |  | ● Comprehensive, interdisciplinary care planning, including preferences and goals of care  ● Regular review of care plan, appropriate dissemination of care plan, initiation of care to address main symptoms | ● |
| Patient centred care and support |  | ● Access to information around services to assist with shared decision making and care planning  ● Care aimed at addressing main symptoms and complaints  ● Social support, Spiritual and existential care, availability of spiritual support and counselling  ● Promotion of advance care planning and completion of advance directives | ● |
| Co-ordination of care |  | ● Communication within day service and between day service and source of referral  ● Agreed care plan available across services | ● |
| Care outcomes and re-assessment |  | ● Assessment of overall quality of life performed using a valid measure  ● Regular review to assess change in main symptoms or care goals in line with the agreed care plan  ● Satisfaction with overall and individual aspects of care  ● Assessment using valid, generic palliative care or condition specific measures to monitor progress | ● |

| **OECD model** | **Effectiveness** | **Access / co-ordination of care** | **Patient-centred care** |
| --- | --- | --- | --- |
| **Living with life-limiting illness**  **Improving / maintaining Quality of Life** | **Main indicators**  ●  ●  ● | **Main indicators**  ●  ●  ●  ●  ● | **Main indicators**  ●  ●  ●  ●  ● |

* Model based on the Donabedian framework [18] and a modified version of the framework used in the OECD Health Care Quality Indicators Project [23]
